# Buttercups and Daisies

**Published:** 1887-06-25

**Authors:** 


### BUTTERCUPS AND DAISIES.

It is a fortunate thing to be bred in the country, whether the child be born poor or rich. Jack Red-poll, aged eight, may possess no clothes but trousers and a shirt, the one out at knees, the other at elbows; and his meat may be as simple as John the Baptist's, except that it is bread and treacle instead of "locusts and wild honey." But master Jack is in no sense an object of pity, at any rate of self-pity. In spite of these and other very evident drawbacks he is generally one of the happiest human beings you will find in a week's march. In the long days of summer every field is his ancestral park, every hedgerow his happy hunting-ground for birds' nests, every stream his flowing fount of water which is better than wine, and his cold bath at the same time, and every wild gooseberry-bush—to say nothing of earthnuts, farmers' orchards, pea-fields, and turnip crops—a well-spread table in which his soul delights.

If Mr. W. H. Mallock were to ask a country lad of this stamp, "Is life worth living?" the lad, if he could express his actual thoughts with precision enough, would probably say, "Are you a lunatic, or are you merely chaffing?" With health and food and sufficient clothing to satisfy the requirements of the parson and the parish beadle, the country lad is happy as a king. The School Board or the National School may have certain transitory terrors for him, and some bigger fellow may be a kind of village Bismarck, whose frown adds wings to the feet; but these are mere sauces which give piquancy to life's daily feast; and until the time arrives for him to go out to service on the farm his life is much more free from cares and fuller of exuberant delights than that of almost any other class of human beings whatsoever.

This is as it should be. All young creatures who are in anything like a natural condition find existence itself a positive delight. The kitten, the lamb, the comical little puppy, the grave colt, and the sedate-looking calf, all manifestly experience a more or less intense pleasure in every part of their lives.

Even to write about these things gives both rest and cheerful visions of a children's Arcadia which is not a mere creation of the fancy. Dr. Jessop may probably think otherwise, but we by no means agree with him. Whatever may be the experience of the rural man and woman, it is undoubted that the average country lad finds life full of keen enjoyment. Youth has a clear title to such enjoyment. As manhood and womanhood advance, not pleasure but duty should be the object of life. The child, however, ought not to be overburdened with the sense of duty, or with those daily tasks which a hard necessity may put upon him. We would not desire to remove from grown-up people the consciousness of struggle and conflict. It is clear that "the constitution and course of nature," as Butler has it, demand of men and women a probationary wrestling with destiny, which in every case must end in victory or defeat. But no man or woman who has thought out the problems of life for himself wishes mere children to experience this consciousness of battle and probation, at any rate in any considerable degree. Life is naturally delightful to them, and if in any State or country a large proportion of children are unhealthy or unhappy, that State and Government must be considered a failure to the extent to which it fails to secure their happiness and health. Of course, we are well aware that many a so-called statesman would pooh-pooh such a proposition as the exaggerated sentimentalism of a philanthropist. We on our part should affirm of such a man, not that he had a hard, practical, and unsentimental head and a cold heart, but that he had a shallow, feeble brain and no eyes.

What constitutes the prosperity of a State? Does it consist in a few rich, self-indulgent, and pleasure-loving people; or is it not rather a country in which the bulk of the population are industrious, virtuous, and moderately contented? Above all, should not the children in any country have at least as much enjoyment as those creatures who are domesticated for man's convenience, or which run wild in the woods and wastes?

Town-bred children no doubt have their pleasures, even among the poorest classes. There is such a natural exuberance about young life that it will triumph over almost any kind of depressing circumstances in which it may be placed. Still, the town-bred child, however fortunate may be his lot, is incomparably poorer in delights than the child of the country. What is a walk in the park with the nurse, or the sailing of a boat on the Round Pond among hundreds of other children, compared with a whole long summer in the woods, by the running brooks, among the flowers, in the hayfield, in the cornfield, in the orchard, in the garden, birds'-nesting, putting up swings in the trees or in the outhouses, tearing your clothes, getting your feet wet, doing a thousand things which the unhappy little pedant of the town would no more dream of doing than he would of spilling the gravy over his new Eton jacket at dinner, or going to church without his gloves and his tall hat? Poor little mortal: so soon forced to enter upon a dreary life of priggish pedantry, to suit the depraved and ignorant tastes of a conventional and ill-instructed age!

But if the lot of the town child of well-to-do parents is thus comparatively tame and joyless, what is the fate of the poor little creatures who are born and bred in the slums? That fate has been so often described and even witnessed by many of the best of men and women, that we will not here attempt to produce a sensational picture which can serve no good purpose. If we had the pen of a Hood or the pencil of a Cruikshank we could convey no adequate idea of the utterly unchildlike and pathetic condition of thousands and tens of thousands of the child-dwellers in the indescribable and heart-breaking slums of our largest towns. The State is able to do little for the happiness of children, but kind-hearted men and women, who in many cases are quite unknown to fame, are both able and willing. Many are the schemes afoot in London and other large centres of population for giving the little denizens of the slums one, two, or even three weeks of real happiness among the green fields. But however numerous may be those schemes, and however kind and generous their promoters, only the smallest tithe of the tens of thousands of London children will spend a week or two among the buttercups and daisies this summer. How glad must be the hearts of those benevolent people who can spare five or ten guineas for so good and blessed an object! Mr. Lawson's idea of giving thousands of small Londoners a Jubilee treat on an unexampled scale was excellent; and although accompanied by great risks, the experiment deserved to succeed. But a day in Hyde Park with thirty thousand others is as nothing compared with two or three weeks among the pure delights of the distant fields and woods. If any of our readers would earn for themselves a lasting sensation of pleasure unalloyed, let them personally join and actively help some of the many movements now afoot for giving the pale-faced and narrow-chested children of the towns two or three weeks in the country. The Rev. S. A. Barnett, St. Jude's, Whitechapel, or Mr. C. S. Lock, County Holidays Fund, 15, Buckingham Street, Strand, W.C., will be glad to hear from any such active helpers.

